# Transcriptome of the Invasive Brown Marmorated Stink Bug, *Halyomorpha halys* (Stål) (Heteroptera: Pentatomidae)

**DOI:** 10.1371/journal.pone.0111646

**Published:** 2014-11-11

**Authors:** Michael E. Sparks, Kent S. Shelby, Daniel Kuhar, Dawn E. Gundersen-Rindal

**Affiliations:** 1 USDA-ARS Invasive Insect Biocontrol and Behavior Laboratory, Beltsville, Maryland, United States of America; 2 USDA-ARS Biological Control of Insects Research Laboratory, Columbia, Missouri, United States of America; The Ohio State University/OARDC, United States of America

## Abstract

*Halyomorpha halys* (Stål) (Heteroptera: Pentatomidae), the brown marmorated stink bug, is an invasive agricultural and nuisance pest rapidly expanding its incidence in North America. This voracious pest poses a significant threat to rural and urban agriculture, especially to specialty crops such as apples, grapes and ornamentals, as well as staple crops including soybean and corn. The object of this study was to generate transcript sequence resources for *H. halys*. RNA-seq libraries derived from distinct developmental stages and sexes were sequenced and assembled into 248,569 putatively unique transcripts (PUTs). PUTs were segmented into three disjoint tiers of varying reliability, with 4,794 classified as gold tier (highest quality), 16,878 as silver, and 14,357 as bronze. The gold-tier PUTs associated with 2,580 distinct non-redundant protein sequences from the NCBI NR database—1,785 of these (69%) mapped to annotated UniProtKB database proteins, from which 1,273 unique Pfam families and 459 unique Molecular Function GO terms were encountered. Of the silver tier's 6,527 PUTs associated with unique proteins, 4,193 mapped to UniProtKB (64%), from which 1,941 and 640 unique Pfam and Molecular Function GO terms were extracted. *H. halys* PUTs related to important life processes like immunity, endocrinology, reproduction, development, behavior, neurotransmission, neurotoxicity, olfaction, and small RNA pathways were validated through quantitative Real-Time PCR (qRT-PCR) for differential expression during distinct life stages (eggs, 2^nd^ instar nymphs, 4^th^ instar nymphs, female adults, male adults). PUTs similar to hypothetical proteins identified in symbiont microbes, including *Pantoea* and *Nosema* species, were more abundantly expressed in adults *versus* nymphs. These comprehensive *H. halys* transcriptomic resources can be utilized to aid development of novel control methodologies to disrupt life processes; to conduct reverse genetic screens to determine host gene function; and to design environmentally unobtrusive means to control host populations or target specific *H. halys* life stages, such as molecular biopesticides.

## Background

*Halyomorpha halys* (Stål) (Heteroptera: Pentatomidae), the brown marmorated stink bug (BMSB), is an invasive insect native to Asia (China, Taiwan, Korea, and Japan) that has emerged in the last decade as an important major insect pest in the United States, Canada and Europe and has become the top invasive insect research priority for the USDA Agricultural Research Service. *H. halys* is a polyphagous piercing/sucking feeder having over 300 known plant hosts. It poses a considerable ecological and economic threat—tens of billions of dollars annually—to specialty crops such as apples, stone and pome fruits, grapes, ornamental plants, vegetables, seed crops, as well as such staple crops as soybean and corn. *H. halys* has rapidly expanded its range from the original single point of accidental introduction and establishment in the Allentown, Pennsylvania area in the late 1990s [1]. Damage has been particularly extensive in the U.S. Mid-Atlantic Region (DE, MD, PA, NJ, VA, and WV) and this voracious pest continues to spread. Currently, *H. halys* has been detected in 40 states and Canada [2], as well as Europe [3]. In addition to its status as a major invasive agricultural pest, *H. halys* is also considered a structural nuisance pest as it invades homes and indoor spaces in high numbers in fall to overwinter as adults before re-emerging in spring [2].

The threat to agriculture from spread of *H. halys* populations has continued to increase, spurring development of management tools; these include traditional classical biological control strategies using natural enemies or predators [2], as well as development of novel management tools including pheromone lures for monitoring and trapping [4], [5], and newer biologically- and genetically-based control methods employing entomopathogenic [6] or molecular [7] biopesticides. The characterization of *H. halys* genomic and transcriptomic sequence data has been needed to aid development of novel, specifically-targeted, effective and environmentally sound means to mitigate the extensive damage produced by this insect, and to slow its aggressive geographic expansion. Because *H. halys* represents one of a broad group of agriculturally significant, highly invasive stink bug pests of the insect family Pentatomidae—which include southern green stink bug (*Nezara viridula*), green stink bug (*Chinavia hilaris*) [8], brown stink bug (*Euschistus servus*), and red-banded stink bug (*Piezodorus guildinii*), to name a few— the *H. halys* transcriptome is needed as a reference transcriptome for genetic marker development and comparative analyses of the functional array of transcripts deployed by this class of insects. The current study obtained a comprehensive RNA-Seq transcriptome dataset from four distinct *H. halys* developmental life stages (2^nd^ instar nymphs, 4^th^ instar nymphs, adult females, adult males); a set of high quality *H. halys* gene structures was delineated and functionally annotated, and the transcriptome-level activity at each developmental stage was assessed and compared. Differentially expressed transcripts were further validated across developmental stages by quantitative real time PCR (qRT-PCR) analyses to gain insight into stage-specific genes that could serve as targets for improved biopesticides. In addition, these *H. halys* transcript resources will be critical for annotation of the *H. halys* genome (currently in process), as well as enabling identification of inherent defense mechanisms against proposed control measures, the ability to defend against entomopathogens, and mechanisms that may enable resistance development.

## Results and Discussion

### Qualitative analysis of *H. halys* RNA-Seq data

**Figure 1:**
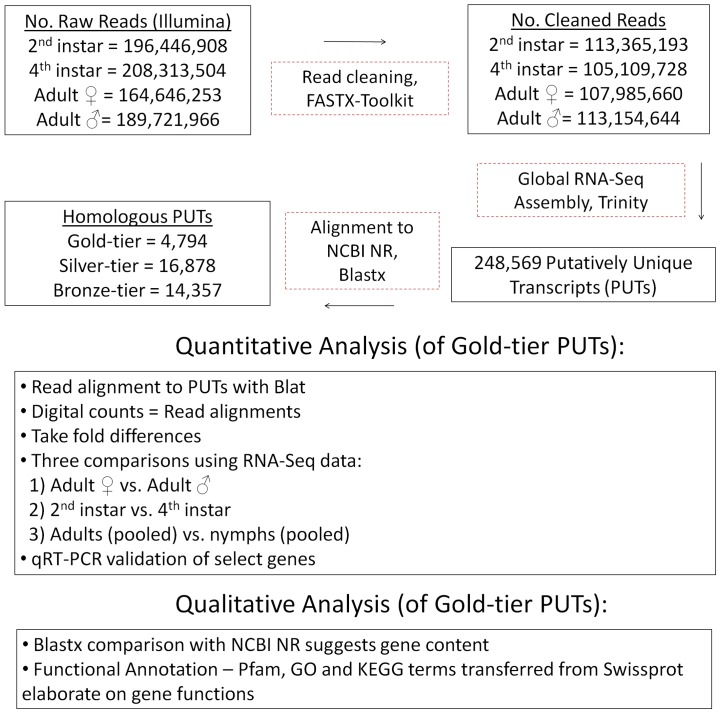
RNA-Seq Assembly and Analysis Protocol. Following data cleaning and assembly into putatively unique transcripts (PUTs), the *H. halys* gene space captured by these data was functionally annotated using information collected from the highly reliable Swiss-Prot subset of UniProtKB. RNA-Seq data was also used quantitatively, in order to identify potential differentially expressed genes for further validation using qRT-PCR assays.

**Figure 2:**
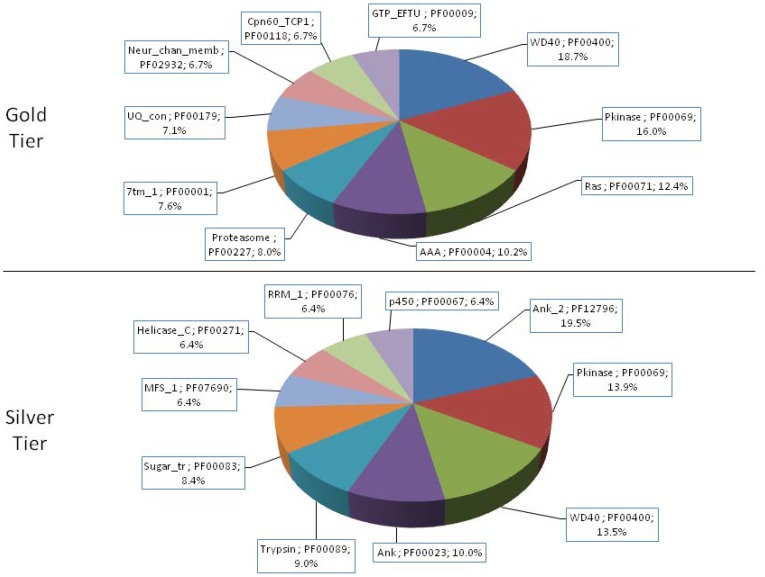
Relative Frequencies of the Ten Most Abundant Pfam Families. For each of the gold and silver PUT tiers, the ten Pfam families most frequently encountered among associated Swiss-Prot exemplars, and their relative abundances, are indicated.

**Figure 3:**
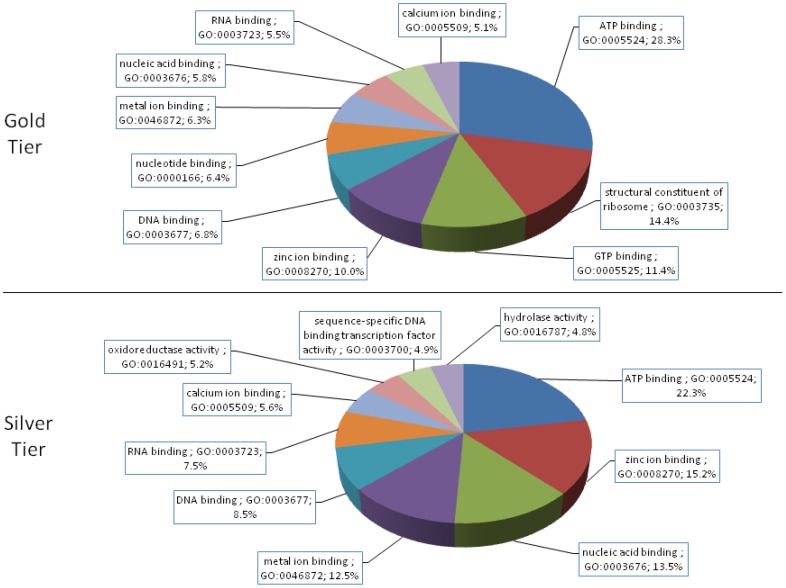
Relative Frequencies of the Ten Most Abundant (Molecular Function) GO Terms. Terms obtained from the Molecular Function aspect of the Gene Ontology were analyzed for their frequency of occurrence in this PUT dataset. The ten terms most frequently encountered among associated Swiss-Prot exemplars, and their relative abundances, are presented.

Figure 1 shows the RNA-Seq assembly and analysis pipeline used in this study, which is further detailed in Materials and Methods. The 439,615,225 RNA-Seq reads surviving quality control procedures were assembled into a total of 248,569 putatively unique transcripts (PUTs). Of these, 4,794 were classified into the gold tier, 16,878 into the silver, and 14,357 into the bronze. The gold-tier PUTs associated with 2,580 distinct non-redundant protein sequences obtained from the NCBI NR database—1,785 of these NR proteins (69%) could be mapped to annotated proteins in the Swiss-Prot subset of the UniProtKB database, from which 1,273 unique Pfam families and 459 unique GO terms from the Molecular Function aspect were encountered. (Gold-tier PUT sequences and their associated annotations are available in Table S1.) The process was similarly repeated for the silver-tiered data: Of the silver tier's 6,527 unique GI numbers, 4,193 mapped to UniProtKB entries (64%), from which 1,941 and 640 unique Pfam and Molecular Function-specific GO terms were extracted. Figures 2 and 3 illustrate the relative abundances of the ten most frequently encountered Pfam family definitions and GO Molecular Function terms encountered in this data set, respectively.

A total of 31 genes were apparently highly differentially expressed, exhibiting a 20-fold or greater difference in expression levels in the 2^nd^-instar *versus* 4^th^-instar comparison. In the nymphs *versus* adult comparison, 41 genes were highly (20+ fold) differentially expressed; in the nymphs *versus* adults comparison, 150 genes were highly differentially expressed. Table 1 shows the five genes having the greatest level of perturbation for each of the three comparisons made, and Table S1 shows RNA-Seq-inferred fold differences in gene expression as observed for all genes.

**Table 1:** *Halyomorpha halys* genes having the most pronounced expression level perturbations as suggested by fold changes in RNA-Seq read abundances.

4th Instar expression relative to 2nd Instar				
FoldDiff	Direction	2nd Instar	4th Instar	NRgene
110.55	up	1.76E-08	1.95E-06	ref|XP_001656025.1| tubulin beta chain
74.06	up	2.65E-08	1.96E-06	dbj|BAB88643.1| platyfish HSP70-1
63.36	up	3.53E-08	2.24E-06	ref|ZP_06714235.1| GTP-binding protein YchF
61.48	up	8.82E-09	5.42E-07	gb|AFP62029.1| Phosphoenolpyruvate carboxykinase
58.24	up	9.70E-08	5.65E-06	gb|AAM33784.1| ribosomal protein S12

Female expression relative to Male				
FoldDiff	Direction	Male	Female	NRgene
24,885.78	down	2.30E-04	9.26E-09	dbj|BAB88643.1| platyfish HSP70-1
23,383.69	down	2.17E-04	9.26E-09	ref|XP_001656025.1| tubulin beta chain
22,865.49	down	2.12E-04	9.26E-09	ref|XP_002731505.1| GL12416-like
10,414.30	down	4.82E-04	4.63E-08	ref|XP_003708589.1| tubulin alpha chain-like
8,891.39	down	1.65E-04	1.85E-08	gb|AFP62029.1| Phosphoenolpyruvate carboxykinase

Nymph expression relative to Adult				
FoldDiff	Direction	Adult	Nymph	NRgene
1,821.93	down	2.67E-04	1.46E-07	pdb|2JNF| Fly Troponin C, Isoform F1
1,668.64	down	7.64E-06	4.58E-09	gb|EFR25008.1| AND_10038, hypothetical
1,110.95	down	1.02E-05	9.15E-09	ref|XP_002424596.1| protein kinase subunit
1,083.78	down	4.96E-06	4.58E-09	ref|XP_002971910.1| SELMODRAFT_172529
657.97	down	3.01E-06	4.58E-09	ref|NP_001155541.1| density-regulated protein

Results for each of the three comparisons performed are presented.

### qRT-PCR-based validation of *H. halys* expressed transcripts

**Figure 4:**
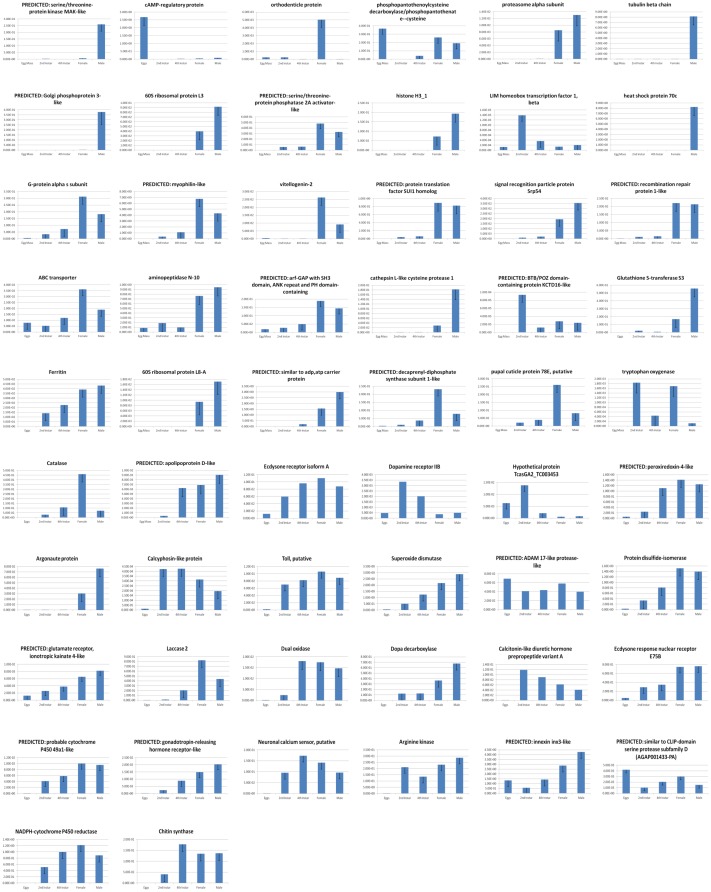
qRT-PCR transcript validation results for *H. halys*. Three biological replicates with three technical replicates each were performed. Bar height denotes the mean average of sample-specific 2^-ΔCt^ values, and error bars represent the standard error of the mean. Thirty-two selected transcripts are shown including certain genes predominantly expressed in adult males (e.g., Golgi phosphoprotein 3-like and tubulin beta chain); predominantly expressed in adult females (e.g., orthodonticle proteins, vitellogenin-2, decaprenyl diphosphate synthase subunit 1-like); predominantly expressed in nymphs (e.g., LIM-homeobox transcription factor beta, BTB/POZ domain containing protein KCTD16-like); and those differentially expressed across various life stages (e.g., apolipoprotein D-like, neuronal calcium sensor). The transcript identified as TcasGA2_TC003453 associates with the NCBI NR protein having GI no. 270004135.

Numerous *H. halys* differentially expressed transcripts were associated with functions in critical life processes, including immune response, endocrinology, reproduction, growth, development, behavior, neurotransmission, neurotoxicity, olfaction, detoxification, insecticide resistance, and other categories. Individual *H. halys* transcripts examined using gene-specific primers (detailed in Table S2) displayed a variety of interesting differential expression patterns observed in males *versus* females and across discrete developmental stages, and these correlated well to initial RNA-seq profiles. For example, various tubulin-related and heat shock protein genes were found to be more highly expressed in males *versus* females; females had higher expression levels of orthodenticle; the egg-related gene, vitellogenin-2; and a decaprenyl diphosphate synthase subunit 1-like gene associated with coenzyme Q production [9]. A LIM homeobox transcription factor beta gene was found preferentially expressed in early nymphal stages (Figure 4). Most patterns were expected; for example, vitellogenin encodes the vitellin protein present in oocytes, and is expressed as an egg yolk precursor in female insects and in the hemolymph of drones and sterile worker bees, as well as in other insects [10], [11]. LIM homeobox transcription factor beta, highly expressed in early instar nymphs, is an evolutionarily conserved gene important in gene regulation during embryonic and early stages of development [12], particularly limb development. The phosphopantothenoylcysteine decarboxylase/phosphopantothenate-cysteine ligase, which functions in coenzyme A biosynthesis, was preferentially highly expressed in *H. halys* eggs and appears to have originated from gammaproteobacteria. The high levels of expression in eggs with substantial expression in adult females suggest this could be associated with symbionts vertically transmitted to eggs by adult females [13], [14].

### Detection of abundant symbiont-associated *H. halys* transcripts: *Pantoea*, *Nosema*, and other spp. and a novel iflavirus

**Figure 5:**
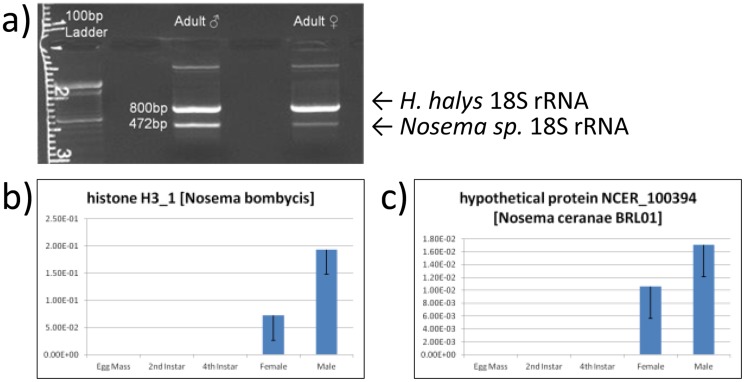
Identification and Quantification of *Nosema* sp. Gene Expression in *H. halys*. A) PCR confirmation of a *Nosema* sp. 18S rRNA gene from cDNA. Amplicon sequencing confirmed a *Nosema* origin of the 472bp band (data not shown). B) qRT-PCR amplification of a histone protein gene similar to a homologous instance from *Nosema bombycis*. C) qRT-PCR amplification of a protein similar to a hypothetical protein identified in *Nosema ceranae*.

A large percentage of abundantly expressed *H. halys* transcripts originated from apparent microbial organisms, including *Pantoea* and *Nosema* species as well as other microbes (*Nosema* transcripts detailed in Tables 1 and 2, additional microbial transcripts detailed in Table S3). This was not surprising based on recent evidence highlighting the importance of gut symbionts in the overall survival and success of *H. halys* in the field [13]: Surface sterilization of eggs to remove vertically transmitted gut colonizing bacteria (primarily *Pantoea agglomerans*) resulted in smaller clutches in the first generation and dramatically reduced survivorship in the second generation. Bansal et al [14] recently examined the gammaproteobacterial endosymbiont harbored in midgut gastric caeca and identified *Candidatus* “Pantoea carbekii”, a close relative of *P. agglomerans*, as the primary bacterial symbiont of *H. halys* based on 16S rRNA sequences. In the present *H. halys* transcriptome data, more than 50 PUTs similar to hypothetical proteins from various *Pantoea* species were identified and were more abundantly expressed in adults *versus* nymphs (nymphs: 2^nd^ and 4^th^ instars combined). Over 20 PUTs similar to hypothetical proteins of *Nosema ceranae*, a honeybee parasite, were also more abundantly expressed in *H. halys* adults *versus* nymphs, as well as PUTs homologous to ribosomal and histone proteins identified in the silkworm parasite, *Nosema bombycis* (Table 2 and Table S1). PCR amplification of 18S rRNA confirmed presence of a novel *Nosema* species in *H. halys* adults—these genes were preferentially expressed in males relative to females (Figure 5). Interestingly, several *H. halys* individuals field collected in Maryland did not display detectable *Nosema* species infections (data not shown), which may indicate the Beltsville lab colony is distinct in this regard. No *Wolbachia*-associated transcripts were identified in the *H. halys* transcriptome, nor were any detected by DNA PCR using *Wolbachia*-specific primers (data not shown), though *Wolbachia* have been observed in association with *H. halys* [13]. Finally, by similar comparative PUT analyses and methodologies, a novel RNA iflavirus was recognized and discovered as being associated with *H. halys*; this iflavirus was the subject of a prior publication [15].

**Table 2:** Fold changes in RNA-Seq read abundances for six representative homologs of various *Nosema* genes identified in the *Halyomorpha halys* transcriptome data.

4th Instar expression relative to 2nd Instar				
FoldDiff	Direction	2nd Instar	4th Instar	NRgene
0.00	up	0.00E+00	9.51E-09	gb|ACJ24176.1| histone H2A [Nosema bombycis]
0.00	up	0.00E+00	9.51E-09	gb|ACU00734.1| 60S ribosomal protein L3 [Nosema bombycis]
0.00	up	0.00E+00	9.51E-09	gb|ADZ95692.1| 60S ribosomal protein L4 [Nosema bombycis]
1.08	up	8.82E-09	9.51E-09	Ref|XP_002995330.1| NCER_101816 [Nosema ceranae]
0.00	up	0.00E+00	9.51E-09	Ref|XP_002995933.1| NCER_101048 [Nosema ceranae]
0.00	up	0.00E+00	4.76E-08	Ref|XP_002995188.1| NCER_102023 [Nosema ceranae]

Female expression relative to Male				
FoldDiff	Direction	Male	Female	NRgene
4.19	down	3.49E-06	8.33E-07	gb|ACJ24176.1| histone H2A [Nosema bombycis]
2.01	down	2.51E-06	1.25E-06	gb|ACU00734.1| 60S ribosomal protein L3 [Nosema bombycis]
3.18	down	2.65E-06	8.33E-07	gb|ADZ95692.1| 60S ribosomal protein L4 [Nosema bombycis]
2.12	down	8.74E-06	4.12E-06	Ref|XP_002995330.1| NCER_101816 [Nosema ceranae]
3.51	down	2.83E-06	8.06E-07	Ref|XP_002995933.1| NCER_101048 [Nosema ceranae]
2.11	down	8.97E-06	4.25E-06	Ref|XP_002995188.1| NCER_102023 [Nosema ceranae]

Nymph expression relative to Adult				
FoldDiff	Direction	Adult	Nymph	NRgene
479.15	down	2.19E-06	4.58E-09	gb|ACJ24176.1| histone H2A [Nosema bombycis]
413.95	down	1.89E-06	4.58E-09	gb|ACU00734.1| 60S ribosomal protein L3 [Nosema bombycis]
385.30	down	1.76E-06	4.58E-09	gb|ADZ95692.1| 60S ribosomal protein L4 [Nosema bombycis]
708.36	down	6.48E-06	9.15E-09	Ref|XP_002995330.1| NCER_101816 [Nosema ceranae]
402.09	down	1.84E-06	4.58E-09	Ref|XP_002995933.1| NCER_101048 [Nosema ceranae]
291.25	down	6.67E-06	2.29E-08	Ref|XP_002995188.1| NCER_102023 [Nosema ceranae]

### Immunity-related transcripts identified in *H. halys* non-induced and immune-induced transcriptome

**Figure 6:**
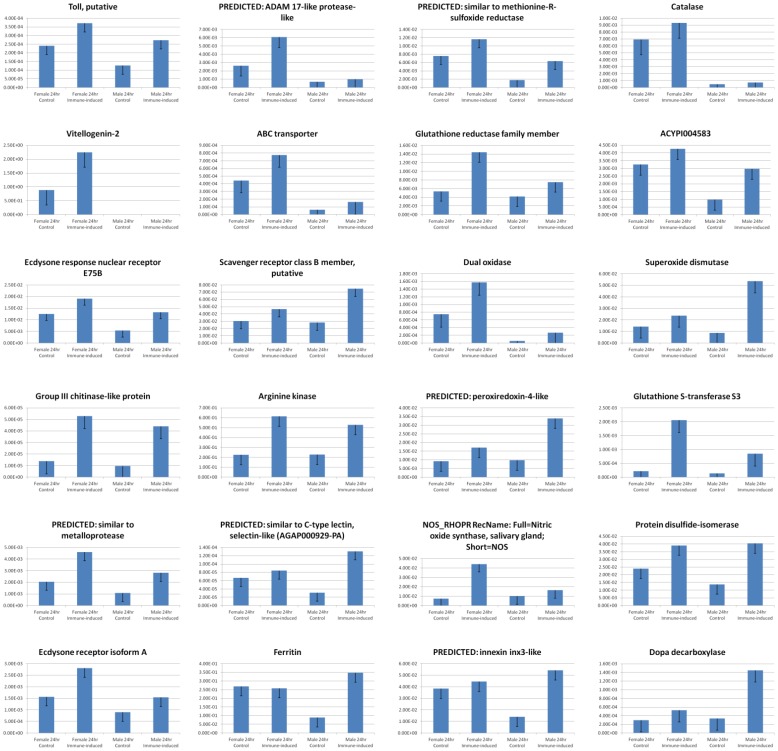
qRT-PCR Transcript Validation Results for Select Transcripts of Immune-Induced Adult Male and Adult Female *H. halys* 24 Hours After Septic Puncture. Three biological replicates with three technical replicates were performed. Bar height denotes the mean average of sample-specific 2^-ΔCt^ values, and error bars represent the standard error of the mean. Twenty-eight selected transcripts are shown including those associated with immune response (e.g., toll, vitellogenin, ecdysone response nuclear receptor, group III chitinase-like, metalloprotease, ecdysone receptor isoform A, DOPA decarboxylase, Adam 17-like, ABC transporter, scavenger receptor class B, ACYP1004583, arginine kinase, C-type lectin, ferritin, innexin); energy metabolism (e.g., methionine-r-sulfoxide reductase, glutathione reductase family, dual oxidase, peroxiredoxin-4-4like, arginine kinase, glutathione-s-transferase, protein disulfide isomerase); and the oxidative stress response (e.g., Met-r-sulfoxide reductase, catalase, dual oxidase, peroxiredoxin-4-4like, nitric oxide synthase, superoxide dismutase, ferritin). The transcript identified as ACYPI004583 associates with the NCBI NR protein having GI no. 239791403.

Insects have complex immune systems that enable them to defend against infections by microbial pathogens. These immune responses are critical to insect survival and must be overcome to effect biological control; thus immune-related PUTs were examined in detail for *H. halys*. All key components of the innate insect immune response were identified in *H. halys*, including core immune signaling, cellular response/immobilization, and RNA interference pathways (discussed separately below). The immune response is triggered in response to systemic infection by pathogen-associated molecular patterns (PAMPs) such as bacterial cell wall or flagellar components in the presence of host damage-associated molecular patterns and chemokines [16]. Pattern recognition receptors located on hemocytes and other tissues bind PAMPs, activating signal transduction pathways that induce antimicrobial peptide biosynthesis. Insects possess an innate immune defense separated into humoral and cell-mediated responses, which may be constitutive or induced following microbial incursion. Though no attempt was made in this study to induce the *H. halys* immune response with microbial elicitors, it was expected that some constitutively expressed components would be present. Surprisingly, several inducible response components were noted in non-immune-stimulated *H. halys* (Figure 4). It is possible that expression of inducible immune pathway gene components is elevated in response to the possible presence of microbial pathogens, including species from the genus *Pantoea* (summarized in Tables 1 and 2, as well as Table S3). Among the PAMPS, pattern recognition receptors, and immune-associated PUTs expressed in the non-immune-induced *H. halys* transcriptome were peptidoglycan recognition protein S2, peptidoglycan-recognition protein precursor, and pattern recognition serine proteinase precursor. Increased expression of selected putative immune-related PUTs was subsequently confirmed by qRT-PCR of immune-induced male and female adults 24 hours following immune induction by non-sterile septic puncture (Figure 6). Proteins responsible for immune recognition of foreign glycoproteins were identified, including C-type lectins, hemocytin, scavenger receptor, and leucine-rich repeat proteins. C-type lectin was up-regulated 4.2-fold and a scavenger receptor PUT up-regulated 2.6-fold in immune-induced adult males (Figure 6).

Pathogen invasion activates core innate immune signaling pathways, including Toll, Imd, and JAK/STAT [17]. These pathways effect the production of a suite of antimicrobial peptides, enzymes, and inhibitors that limit growth of pathogens [17]. *H. halys* PUTs homologous to Toll pathway components included nuclear factor kappa-B kinase, spaetzle, spaetzle 4, spatzle 5, dorsal and snake. PUTs homologous to antibacterial peptides of other insects identified included an antibacterial peptide homolog of *Tribolium castaneum* hyphantrin and a lysozyme transcript—these are normally expressed at extremely low levels in uninfected insects, but were relatively abundant in *H. halys*. Immune stimulation slightly up-regulated a Toll-like PUT by 1.2-fold in adult males and 1.5-fold in females (Figure 6). Within Heteroptera, the model insect *Rhodnius prolixus*, closely related to *H. halys*, has been shown to possess components of the insect signal transduction eicosanoid pathway interacting with trypanosomal and bacterial infection [18], [19]. Homologs of eicosanoid pathway components were noted in the *H. halys* non-induced transcriptome assembly, including a calcium-independent phospholipase A2 enzyme, phospholipase A2-activating protein, 15-hydroxyprostaglandin dehydrogenase, prostaglandin E synthase, prostaglandin GH synthase, and prostaglandin reductase.

Many constitutively expressed proteins related to the cellular mobilization response (phagocytosis, autophagy, melanization, and coagulation) were sampled in *H. halys*, such as homologs of cadherin, annexin, cell adhesion molecule 3, hemicentins and beta-integrins. PUTs encoding an engulfment and cell motility protein and lingerer were identified. A PUT corresponding to an insect gap junction protein, innexin, was identified, suggesting monomers involved in gap junction formation following cellular activation were present; this innexin was up-regulated 3.9-fold in immune-stimulated males, but was not up-regulated in immune-stimulated females (Figure 6). Components of the melanization reaction, which is catalysed by phenoloxidase and initiates synthesis of melanin, leading to crosslinked proteins and cytotoxic free radicals [17], [20], [21], were numerous. Transcripts corresponding to prophenoloxidase, putative serpins, and serine proteases of the prophenoloxidase regulatory cascade were recognized: these included a full length homolog of prophenol oxidase subunit 2, as well as prophenoloxidase activating factor, serpins, clip-domain serine protease, and hemolymph proteinase-5. Clip-domain serine protease expression was increased 3.2-fold as a result of activation of the immune response (Figure 6). Additional melanization pathway enzymes such as DOPA decarboxylase, dopamine N-acetyltransferase, and dopachrome conversion enzyme, and laccases were up-regulated; DOPA decarboxylase was increased 1.8-fold in females and 4.3-fold in immune-stimulated males (Figure 6). Additionally laccase, multicopper oxidases, Ebony, and proteins associated with cuticular melanization and coloration were identified, with laccase transcription elevated 3.1-fold in immune-stimulated males (Figure 6). Phenoloxidase-activated hemolymph coagulation defence pathways are utilized against multicellular invaders [22]. Components of these pathways were identified in *H. halys*, including transcripts encoding anticoagulant serine protease, clotting factor B-like, coagulation factor XI, proclotting enzyme-like, limulus clotting factor c and a multiple coagulation factor deficiency protein.

Cytotoxic reactive oxygen and nitrogen species generated by hemocytes and other tissues elicit a non-specific defense against all invaders [23]. *H. halys* PUTs similar to the plasma membrane reactive oxygen generator NADPH oxidase, which drives the respiratory burst phenomenon [24], and nitric oxide synthase, an antimicrobial free nitrogen radical generator [25], were identified. Enzymes responsible for inactivation of reactive oxygen species—for example, superoxide dismutase (up-regulated 1.7-fold in immune-stimulated females; 6.2-fold in males) and catalase (up-regulated 1.4-fold in immune-stimulated males and females)—were identified. Dual oxidase transcripts were elevated 2.1-fold in females, and 4.8-fold in immune-stimulated males (Figure 6). A transcript encoding the iron storage and transporting protein ferritin was up-regulated 3.9-fold in immune-stimulated males (Figure 6).

Up-regulation of arginine kinase transcription suggested that immune stimulation altered energy metabolism. Arginine kinase levels were elevated 2.3-fold and 2.7-fold, respectively, in immune-stimulated males and females (Figure 6).

### Transcripts Related to Endocrinology, Reproduction, Growth and Development

Both male and female adults were sampled separately, resulting in identification of assemblies homologous to reproduction-related transcripts of other insects. Proteins required for vitellogenesis, follicle and egg maturation–including vitellogenin and vitellogenin carboxypeptidases, vitelline membrane proteins, and chorion peroxidases–were identified. qRT-PCR results demonstrated differential expression of vitellogenin in adult females (Figure 4). This protein is synthesized and secreted by the fat body and shuttled to developing follicles in the ovaries. Vitellogenin transcripts were up-regulated by septic puncture of adult females (Figure 6), which was expected since its increased transcription is a known insect antibacterial response [26]. Transcripts encoding components of the male reproductive system also were noted, among them testis- and spermatogenesis-related proteins; sperm membrane proteins; sperm flagellar components; and ejaculatory and accessory gland proteins comprising proteases and aconitase.

*H. halys* undergoes incomplete metamorphosis, molting through five nymphal stadia to become an adult. Insect molting is rigorously choreographed, regulated by precise timing of release of several peptide hormones, and coordinated fluctuations in the titers of both juvenile hormone and ecdysone. Disruption of the molting process through chemical means, including growth and development hormone mimics, has been a promising method of insect control [27]. Plant-expressed RNAi targeting the insect-specific metamorphosis regulation gene HaHR3 was shown to be lethal in *Helicoverpa armigera* [27]. Thus, acquisition of tools to study this vital life process in *H. halys* was an objective of this study. Inclusion of an RNA pool containing earlier nymphal instars and molting individuals allowed sampling of *H. halys* nymphal and adult cuticular proteins, resulting in the identification of PUTs putatively involved in cuticle assembly, pigmentation and integrity (*e.g*., chitin synthase, chitin deacetylase, chitotriosidase, laccases, cuticulins). Peritrophin and chitin-binding peritrophin precursor PUTs were also identified. Peptide hormones and corresponding receptors utilized during development and molting were recognized in *H. halys*, including prothoracicotropic hormone, allatostatin and its receptor, bursicon, eclosion hormone, and ecdysis triggering hormone precursor. Contigs orthologous to enzymes in the insect steroid synthesis/degradation pathways also were identified (17-β-hydroxysteroid dehydrogenases, 7-dehydrocholesterol reductase, sterol desaturases, and sterol o-acyltransferases) as well as PUTs similar to the isoprenoid juvenile hormone (JH) synthesis and degradation enzymes of other insects, including JH acid methyltransferases, JH epoxide hydrolases, JH esterases, epoxide hydrolases, and farnesoic acid O-methyltransferase. Also present were homologs of known haemocoelic hormone transporters such as lipophorins, JH binding proteins, oxysterol binding proteins and sterol carrier proteins.

Additional peptide hormones and receptors involved in regulation of metabolism were identified, including leptin receptor-like protein, calcitonin and its receptor, cardioacceleratory peptide receptor, gonadotropin-releasing hormone receptor-like, PDGF- and VEGF-related factors, octopamine receptor, retinoid X receptor, and tachykinin receptor. Also present were PUTs for the corticotropin releasing factor/diuretic hormone and CAPA receptor, 5-hydroxytryptamine receptor system regulating diuresis in *R. prolixus* [28]. *H. halys* PUTs for insulin and mTOR pathways involved in energy metabolism included glycogen synthase, glycogen debranching enzyme, glycogen phosphorylase, trehalase, trehalose 6-phosphate synthase, insulin-like peptide, insulin-like peptide receptor, insulin receptor substrate, and insulin-degrading enzyme.

Eggs were included as a developmental stage in qRT-PCR analyses, though sampling all stages of embryonic development was not undertaken. A number of *H. halys* PUTs homologous to developmental proteins identified in other insects were identified, including instances of armadillo, caudal, bicaudal, bicoid, decapentaplegic, mothers against decapentaplegic, and ultrabithorax. Availability of these sequences should facilitate evolutionary developmental (evo-devo) studies using hemipteran bugs as an experimental system.

### Transcripts Related to Behavior, Neurotransmission, Neurotoxicity, and Olfaction

Insect nervous systems have a critical role in coordinating all behaviors, from vision and locomotion, to mating and host plant location. Many PUTs similar to neurotransmitter receptors, G-protein coupled receptors, and enzymes involved in neurotransmitter synthesis/degradation were identified. Resistance to (or detoxification of) insecticides and plant-based toxic secondary metabolites are major contributors to the survival of herbivorous insects. Insect detoxification enzymes include cytochrome P450s and esterases, and many distinct homologs of these were present in *H. halys*. Glutathione S-transferase transcript levels were elevated 9.8-fold in females subjected to septic puncture, and 6.1-fold in males (Figure 6). Numerous multidrug transporters and ABC transporters responsible for xenobiotics efflux were noted. Transcripts encoding genes involved in olfaction and gustation were also present, including odorant binding and receptor PUTs, olfactory receptors, pheromone binding and degrading enzymes, and signal transduction pathway intermediates.

### Transcripts Related to Small RNA Pathways

Components either directly composing or associated with the double-stranded (ds) RNA processing systems required for effective gene silencing in insects were present in *H. halys*. Transcripts encoding proteins involved in dsRNA cleavage and endonuclease activity, including cytoplasmic subunit argonaute-2, piwi, dicer-2, helicases, and RISC-loading complex subunits, were discernable. Interestingly, no *sid* or *sid*-like [29] PUTs were detected; these have been reported in all insect systems (except dipterans) to be involved in initial dsRNA uptake and transport pathways [30]. PUTs for scavenger receptors and lipophorins, thought to have roles in cellular uptake of RNA [31], [32], were found in *H. halys* assemblies.

Insect transcriptomes have become critical for evaluation of differential transcript expression profiles for biotechnological use [33], and a principal motivation in this study was to identify suitable gene silencing targets for ongoing *H. halys* control research efforts. Numerous differentially-expressed or stage-specific PUTs identified constitute excellent targets for further experimentation. For example, dsRNA synthesized from *Anthonomus grandis* chitin synthase 1 gene has been shown in injected cotton boll weevil female adults to silence chitin synthase 1, and has similarly been reported for silencing the laccase gene [33]. Both transcripts are highly and differentially expressed in *H. halys*. Several bacterial symbiont-associated transcripts also represent gene silencing targets, including those associated with symbiont microbial species.

Collectively, the *H. halys* transcriptome identifies a wide variety of differentially expressed transcripts, providing a reliable source of candidate genes involved in key physiological processes. This resource will enable multiple control projects for this insect pest, especially those targeting specific life stages, including identification and characterization of pathological agents with possible utility for the biological control of *H. halys* populations [15] and the identification of novel RNAi targets, including both bacterial symbionts and key *H. halys* transcripts associated with specific life stages or sexes. These sequence resources will also facilitate annotation of the *H. halys* genome, and provide a needed reference for comparative transcriptomics with other species of stink bug pests. Likewise, genes induced in certain *H. halys* life stages (e.g., vitellogenin-2 and tubulin) or associated with specific biological systems (e.g., ecdysone-related peptide binding proteins, laccase, chitin synthase and cytochrome p450) represent key genes for further assessment, and RNAi-based gene knockdown experiments are currently in progress. If they prove to be sufficiently species-specific, these genes could suggest possible targets for RNAi-mediated gene disruption that may be useful towards diminishing this destructive insect pest.

## Materials and Methods

### Insect Rearing and Dissection

*H. halys* (BMSB) insects were reared as previously described [4] in a colony maintained at USDA-ARS in the Beltsville Agricultural Research Center, Beltsville, MD; this colony was established in 2007 from adults collected in Allentown, PA, and supplemented annually with several Beltsville, MD-collected individuals. Briefly, insects were reared in ventilated plastic cylinders (21×21 cm OD) on a diet of organic green beans, shelled sunflower and buckwheat seeds (2∶1, w/w), and distilled water supplied in cotton-stopped shell vials. Eggs were collected weekly, hatched in plastic Petri dishes with a water vial and, after molting to second-instars, nymphs were transferred to larger rearing cages for the remaining four instars. Adults, males and females were separated 1 to 2 days post emergence, and subsequently maintained in different containers. Insects were maintained in Thermo Forma chambers (Thermo Fisher Scientific) at 25°C and 72% relative humidity, under a 16L:8D photoperiod. Insects of all developmental life stages were collected for RNA extraction, including eggs that originated from a single non-washed egg mass collected <20h after laying. Four discrete developmental life stages were selected for transcriptome analyses: 2^nd^-instar nymphs, 4^th^-instar nymphs, adult males and adult females. Adult males and females were seven days old and had reached maturity. A separate set of adult male and female individuals were also immune-stimulated by septic puncture to the ventral side using a non-sterile minutien pin, followed by RNA extraction 24 hours after puncture, to facilitate analyses of immunity-related and other transcripts.

### RNA Extraction

In each of the four developmental life stages characterized, five individuals were pooled and placed in a tube on ice to calm the insects. Tubes were then placed in liquid nitrogen for approximately 30 sec. Denaturing solution provided from the Totally RNA kit (Life Technologies, Grand Island, NY) was added to the tubes totaling 5 ml. Using an ultra-turrax T25, samples were homogenized until all individuals were fully dispersed (1–2 min., depending on insect size) and samples were placed on ice. RNA was extracted immediately thereafter following the Totally RNA protocol. RNAs were aliquoted as 10 µg in 100 µl of DEPC-treated water and stored at −80°C until sent to University of Georgia Genomics Facility for RNA-seq processing.

### RNA-Seq Library Construction and Sequencing

TruSeq RNA libraries were prepared from total RNA. rRNA depletion was performed, and SE100 reads were sequenced from resulting libraries using an Illumina HiSeq 1000 instrument. These data have been made available at the NCBI Sequence Read Archive [SRA BioProject Acc. No. SRP040652].

### RNA-Seq Data

The FASTX-toolkit (http://hannonlab.cshl.edu/fastx_toolkit) was used to clean sequenced reads. The process eliminated artifact reads, and terminal spans of bases having Phred scores less than 21 were clipped, thereby forcing read ends to have error rates of less than 1%. Trimmed reads of less than 36 bases were purged, as were reads for which 10% or more of bases had a Phred score of 20 or less. Among surviving reads, bases having Phred quality scores of less than 21 were masked with the symbol ‘N’. In total, 58% of reads originally sequenced were retained for analysis, as well as 58% of bases originally sequenced (see Table 3).

**Table 3:** Sample-specific and global RNA-Seq data sizes for *H. halys* pre- and post-quality control.

		2nd instar	4th instar	Adult male	Adult female	Total
Raw	Reads	196,439,408	208,313,504	189,721,966	164,646,253	759,121,131
	Bases	19,643,940,800	20,831,350,400	18,972,196,600	16,464,625,300	75,912,113,100
Cleaned	Reads	113,365,193	105,109,728	113,154,644	107,985,660	439,615,225
	Bases	11,304,674,285	10,477,330,878	11,288,454,095	10,774,361,340	43,844,820,598

### Sequence Analysis and *de novo* assembly of transcripts

Figure 1 presents a graphical overview of the RNA-Seq data analysis approach used. Cleaned RNA-Seq reads were globally pooled and assembled into putatively unique transcripts (PUTs) using the Trinity assembler [34]. These were aligned to the non-redundant protein database, NCBI NR, using Blastx [35]. Homology information was used to identify PUTs that were likely non-spurious, and these were partitioned into three mutually exclusive sets on the basis of alignment quality. The gold-tier gene set represented the most highly reliable set of transcripts, and inclusion required that the associated Blastx hit consisted of a single high-scoring segment pair (HSP), that the PUT query was at least 300 bases in length, that the alignment's subject sequence (i.e., an NR protein) was at least 100 amino acid residues in length, that 75% or more of aligned residues were positively similar, and that the ratio of hit length to subject sequence length was at least 90%. Only the top-scoring hit per PUT was considered. End sequences from PUTs that were not incorporated into Blastx-derived alignments were clipped, and the remaining coding sequences were translated into high-quality *H. halys* protein sequences using the EMBOSS package's Transeq utility [36]. For inclusion in the silver-tier gene class, a PUT had to be at least 100 nucleotides in length and exhibit a hit length covering at least 75% of the NR subject sequence's length. Finally, PUTs of the bronze-tier class were required to be at least 100 nucleotides long and have a hit covering at least 30% of the NR protein's length.

### Functional Annotation

To identify functional capacity in the gold-tiered data, GI numbers for these PUTs' best NCBI NR exemplars were mapped to UniProtKB entries using the “ID Mapping” tab from the UniProt website [37]. These mappings enabled the transfer of associated GO, Pfam and KEGG annotation terms from UniProtKB entries onto *H. halys* gold-tier PUTs. Immune-, developmental- and olfaction-related transcripts contained in the silver and bronze data tiers were identified using GO and InterPro descriptors generated with Blast2GO [38]. Significant hits were confirmed by multiple sequence alignment to homologous arthropod transcripts.

### Sample-specific Gene Expression Quantification

Following quantitative protocols established in a previous insect transcriptome investigation [39], RNA-Seq data were used to suggest potentially up- or down-regulated genes among the gold-tier gene set. Three comparisons were performed: 4^th^ instar against 2^nd^ instar, adult female against adult male, and nymph against adult. No pooling of sequencing lanes was necessary for the first two of these comparisons, though for the last, the 4^th^ and 2^nd^ instar nymph data were pooled to create the nymphal representation, and the adult female and male samples were pooled to create the adult test set. To generate sample-specific gene expression levels, unassembled, cleaned reads were aligned to trimmed gold-tier PUT sequences using Blat [40]. An aligned read incremented a PUT's digital expression level if not less than 95% of its length aligned with 100% sequence identity. If a read mapped to multiple PUTs under these criteria, only its highest-scoring alignment was considered. (If multiple alignments shared this highest score, at most one representative PUT was chosen on an arbitrary basis.) In cases where multiple gold-tier PUTs corresponded to the same NR protein—resulting from such phenomena as gene duplication or alternative splicing—then the counts of all such PUTs were summed and attributed to the responsible protein coding gene. Normalization of the resulting digital expression counts was performed by dividing each protein coding gene's absolute digital count by the total number of unassembled, cleaned reads in the sample. Fold differences in relative gene expression levels were ranked to suggest differentially expressed *H. halys* genes. These candidate transcripts were then flagged for further inspection using qRT-PCR.

### Validation of select transcripts using quantitative real-time PCR (qRT-PCR)

A panel of *H. halys* genes having RNA-Seq fold-level changes suggesting significant differential gene expression was statistically validated using qRT-PCR analysis, performed using three biological and three technical replicates. Quantitative real-time expression experiments were conducted using primers designed from PUT templates with PrimerPlex 2.62 (PREMIER Biosoft, Palo Alto, CA); primer sequences are provided in Table S2. In addition to 2^nd^ and 4^th^ instar, adult male and adult female cDNAs, qRT-PCR was performed for an egg mass sample. Reactions were conducted using an ABI 7500 Real Time PCR System (Applied Biosystems, Carlsbad CA). For each replicate, first strand cDNA was synthesized from 1–5 ug RNA using Superscript Reverse Transcriptase II (Life Technologies, Carlsbad, CA). Each qRT-PCR reaction consisted of 6.25 µl of Power SYBR Green PCR Master Mix (Life Technologies, Carlsbad, CA), 50 ng of diluted cDNA and 1 µM of each primer in a total volume of 12.5 µl. Reactions were performed in triplicate to ensure consistent technical replication and run in 96-well plates under the following conditions: 50°C for 2 min, 95°C for 10 min, and 40 cycles of 95°C for 15 sec and 60°C for 1 min. Melting curves (60°C to 95°C) were derived for each reaction to ensure a single product. Relative gene expression was evaluated with DataAssist Software version 3.0 (Applied Biosystems/Life Technologies), using *H. halys* 18S rRNA and the ER-associated endoreticulocalbin housekeeping gene [41] as endogenous controls for RNA load and gene expression, respectively. 18S rRNA is commonly used for control purposes in quantitative eukaryotic transcriptome studies [42], and endoreticulocalbin was bioinformatically identified as a quality expression control gene for *H. halys* normalization—its transcription level was shown to be the most minimally perturbed across all three comparisons and suitable primers could be designed for it using the PrimerPlex software. A basic geometric argument demonstrated its expression consistency: Because fold changes in gene expression levels were calculated by placing the greater abundance level in the ratio's numerator, these ratios are strictly ≥1 for instances in which counts were detected in both samples being compared (i.e., both numerator and denominator were positive-valued). Thus, in three-dimensional space, an idealized reference point denoting an unperturbed transcript is (x,y,z)  = (1,1,1), where x, y and z denote 4^th^ instar *versus* 2^nd^ instar, adult female *versus* adult male, and nymph *versus* adult comparisons, respectively. Reticulocalbin minimized the Euclidean distance from this reference point (data not shown), which was calculated using the equation:
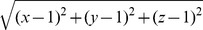
in which variables x, y and z correspond to the absolute fold change values for the three comparisons listed above.

## Supporting Information

Table S1 ***Halyomorpha halys*** **gold-tier PUTs BLAST2GO annotations using the NCBI NR database.**
(XLSX)

Table S2 **Primers designed in this study for** ***Halyomorpha halys*** **qRT-PCR transcript validation, including GenBank gene identifying information for each transcript.**
(XLSX)

Table S3 **Select microbial-origin transcripts identified in the** ***Halyomorpha halys*** **transcriptome that exhibit possible differential gene expression according to fold changes in RNA-Seq read abundances.** Data are presented for three comparisons: 2^nd^ instar *versus* 4^th^ instar, male adults *versus* female adults, and adults (males and females combined) *versus* nymphs (2^nd^ and 4^th^ instar combined).
(XLSX)
